# GMO detection using a bioluminescent real time reporter (BART) of loop mediated isothermal amplification (LAMP) suitable for field use

**DOI:** 10.1186/1472-6750-12-15

**Published:** 2012-04-30

**Authors:** Guy Kiddle, Patrick Hardinge, Neil Buttigieg, Olga Gandelman, Clint Pereira, Cathal J McElgunn, Manuela Rizzoli, Rebecca Jackson, Nigel Appleton, Cathy Moore, Laurence C Tisi, James AH Murray

**Affiliations:** 1 Lumora Ltd, Bartholomew Walk, Cambridgeshire Business Park, Ely, Cambridgeshire CB7 4EA, UK; 2 Cardiff School of Biosciences, Biomedical Sciences Building, Museum Avenue, Cardiff CF10 3AX, UK

## Abstract

**Background:**

There is an increasing need for quantitative technologies suitable for molecular detection in a variety of settings for applications including food traceability and monitoring of genetically modified (GM) crops and their products through the food processing chain. Conventional molecular diagnostics utilising real-time polymerase chain reaction (RT-PCR) and fluorescence-based determination of amplification require temperature cycling and relatively complex optics. In contrast, isothermal amplification coupled to a bioluminescent output produced in real-time (BART) occurs at a constant temperature and only requires a simple light detection and integration device.

**Results:**

Loop mediated isothermal amplification (LAMP) shows robustness to sample-derived inhibitors. Here we show the applicability of coupled LAMP and BART reactions (LAMP-BART) for determination of genetically modified (GM) maize target DNA at low levels of contamination (0.1-5.0% GM) using certified reference material, and compare this to RT-PCR. Results show that conventional DNA extraction methods developed for PCR may not be optimal for LAMP-BART quantification. Additionally, we demonstrate that LAMP is more tolerant to plant sample-derived inhibitors, and show this can be exploited to develop rapid extraction techniques suitable for simple field-based qualitative tests for GM status determination. We also assess the effect of total DNA assay load on LAMP-BART quantitation.

**Conclusions:**

LAMP-BART is an effective and sensitive technique for GM detection with significant potential for quantification even at low levels of contamination and in samples derived from crops such as maize with a large genome size. The resilience of LAMP-BART to acidic polysaccharides makes it well suited to rapid sample preparation techniques and hence to both high throughput laboratory settings and to portable GM detection applications. The impact of the plant sample matrix and genome loading within a reaction must be controlled to ensure quantification at low target concentrations.

## Background

As the world's agricultural systems endeavour to sustain an expanding population, technologies have become available to increase the yield and viability of cultivated crops including the introduction of novel traits into crops using genetic transformation of foreign DNA to produce GM varieties. However, public resistance to commercialization of genetically modified plants is still widespread in Europe [1,2]. Existing European regulation limits the extent of GM presence in non-GM foodstuffs, and the increasing introduction of GM products into Europe is likely to result in parallel GM and non-GM ("conventional") supply chains. In addition, the more widespread planting of GM crops in Europe will lead to the need for on-farm confirmation of GM status. Together these factors are likely to lead to a substantial increase in the extent and frequency of testing for the presence of DNA of a GM-derived origin.

The European Union has currently defined the proportion of GM that can be present to be no more than 0.9% GM in a non-GM product [3-5]. As a consequence, diagnostic tests must be deployed that can accurately quantify the GM proportion for monitoring [6]. Careful sampling and handling techniques are required to ensure the analysis is statistically relevant and appropriate controls are also needed to compare the presence of a transgene to a suitable reference gene.

Several nucleic acid amplification techniques (NAATs) are available for the detection of GM contamination in plants and food [7,8] of which the polymerase chain reaction (PCR) is by far the most widely used. However PCR requires rapid thermo-cycling to denature the target DNA strands, prior to and during amplification [9,10], which imposes specific equipment requirements. Since the discovery of DNA polymerases with strand displacement activity, novel amplification methods have been developed which operate under isothermal conditions (iNAAT) and propagate the initial target sequence by promoting strand displacement using enzymes or modified oligonucleotides.

Loop-mediated isothermal amplification (LAMP) is a sensitive, rapid and specific nucleic acid amplification technology. It is characterized by the use of 4 different primers, specifically designed to recognize 6 distinct regions on the target DNA template, and proceeds at a constant temperature driven by invasion and strand displacement [11-13]. Amplification and detection of target genes can be completed in a single step at a constant temperature, by incubating DNA template, primers and a strand displacement DNA polymerase. It provides high amplification efficiency, with replication of the original template copy 10^9^-10^10 ^times during a 15-60 min reaction [13]. The primer pairs used in LAMP are given specific designations; LAMP primers that generate hairpin loops, the outer displacement primers, and LOOP primers that accelerate the reaction by amplifying from the hairpin previously created by the LAMP primers [13,14].

**Figure 1:**
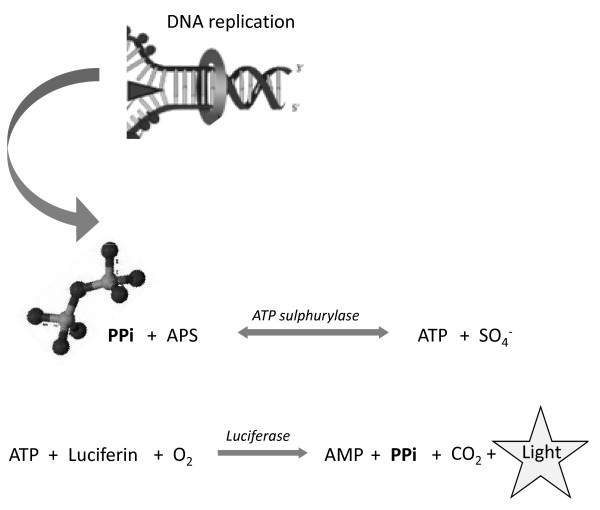
**Chemistry of the BART bioluminescent coupled assay**. PPi liberated during DNA synthesis reacts with adenosine-5'-O-persulfate (APS) in a reaction catalysed by adenosine triphosphate sulfurylase, to form adenosine triphosphate (ATP). A recombinant thermostable firefly luciferase liberates light, CO_2_, PPi and adenosine monophosphate (AMP) in the presence of the substrates luciferin and oxygen.

Several methods exist to determine the extent that DNA has been amplified either after or during a given reaction, of which the most frequently used are the incorporation of fluorescent primers into the amplification product or the use of intercalating fluorescent dyes. Other techniques monitor side products of the DNA synthesis responsible for the amplification reaction. For example, turbidity and fluorescence techniques can also used to detect inorganic pyrophosphate liberated during nucleic acid amplification [15,16]. A recently described bioluminescence real time assay [BART] [17-19] allows the quantitative analysis of iNAATs, in real time. The biochemistry of BART is based on the 'Enzymatic Luminometric Inorganic pyrophosphate Detection Assay, or "ELIDA" [20,21] (Figure 1). Unlike previous applications of the ELIDA assay (most notably Pyro-sequencing™), BART allows dynamic changes in pyrophosphate levels to be monitored continuously in real-time over extended periods at 60°C for up to 2 hours. During a BART reaction, the level of light output increases to a peak whose timing under the same assay conditions reflects the initial concentration of the targeted DNA. Hence quantification of BART reactions utilises the time to peak light output and is not dependent on absolute light intensity produced, which greatly simplifies data interpretation and the hardware requirements, as well as making assays robust to turbidity and suspended solids [19].

The accuracy of molecular diagnostic tests is dependent on appropriate integrity, purity and concentration of the input DNA and therefore on the choice of sample extraction procedure [22-24]. Plant tissues contain a variety of well-known compounds that can be inhibitory to molecular amplifications [25], including acidic polysaccharides, a variety of salts, secondary metabolites and phytochelatins. Most plant genomic DNA extraction technologies are designed to reduce or eliminate these contaminants. Polysaccharides can be removed by exploiting their differential solubilisation in solutions containing detergents, and affinity resins have also been used for the same purpose [26,27]. Hydrophobic cell constituents such as lipids and poly-phenols are routinely excluded from DNA extracts by partitioning with organic solvents, such as chloroform and alcohol. Unfortunately, many of the reagents used to extract and stabilize DNA, such as ethylene diamine tetra-acetic acid (EDTA), phenol, and the ionic detergents, sodium dodecyl sulfate (SDS) and cetyl tri-methyl ammonium bromide (CTAB) also tend to affect NAAT performance [28-31]. Measures to avoid carrying-over these contaminants can make these protocols labour intensive and time consuming to yield DNA of a sufficient quality for PCR.

Several published reports demonstrate that LAMP amplifications tolerate higher levels of certain inhibitors than PCR [32-34]. This suggests that LAMP could have a capacity to amplify polynucleotides from rapidly processed and crude sample matrix derived from plant material [34,35]. Other factors that affect the reliable detection and quantification of low target copy polynucleic acids using this technique are likely to include overall DNA loading within a reaction, which can have a impact upon sensitivity, as it possibly influences non-specific primer interactions [36]. Hence genome size, ploidy and unknown sources of contaminating DNA could affect amplification performance by altering the ratio of target to non-target DNA presence and hence potentially making target quantification and comparisons with reference samples and standards inaccurate. Here we demonstrate the use of LAMP-BART to detect GM events at low copy number levels in samples derived from maize, which has a large genome size and hence a relatively high proportion of non-target DNA. We show that LAMP-BART tolerates crude plant extracts without significant inhibition and examine the characteristics of the sample matrix that impact upon the quantitative nature of this technique and demonstrate its suitability in fieldable systems.

## Methods

### Plant material

Wild-type (Pure Gold) and transgenic (Mon810) maize were grown in 4 inch pots containing Sinclair Multipurpose compost for 4 weeks in a glasshouse that maintained a temperature of 25°C and a 16-h photoperiod, supplemented when necessary to a photon flux density of 350 μ mol m^-2 ^s^-1^. Analysis of GM reference samples were performed on blends of lyophilized powdered Bt11 maize and Roundup Ready Soya (European Reference Material; with GM contents verified to be 0, 0.1, 0.5, 1, 2 & 5% w/w).

### Conventional Genomic DNA extractions

40 mg of lyophilized or 200 mg fresh tissue were extracted using the Genome Wizard kit (Promega), Nucleon Phytopure kit (GE Healthcare) both according to the manufacturer's instructions, or the CTAB (cetyl trimethyl ammonium bromide) extraction method, which included RNase and proteinase digestions [37]. Once extracted the genomic DNA was resuspended in 100 μl of 100 mM DNAse-free Tris-HCl (pH 8.0) and refrigerated in non-stick plastic micro tubes (Ambion; Life Technologies) until required for analysis.

### Rapid genome extraction

50 mg of fresh or lyophilized plant material was ground in 500 ul of genome extraction buffer (700 mM NaCl, 5% Chelex dissolved in 10 mM Tris-HCl buffer; pH 8). The extract was maintained at 100°C for 10 min, mixing regularly. 100 μl of the boiled extract was then desalted using a 0.5 ml Zeba column (Pierce; pre-equilibrated with 3 washes of Tris-EDTA buffer, pH 8), using a syringe to displace the DNA. The final elute contained the partially purified genomic DNA extract suitable for LAMP-BART reactions.

#### DNA quantity and purity

Genomic DNA was quantified by measuring the sample absorbance between 230 and 300 nm on a NanoDrop spectrophotometer. 1 μl of each DNA sample was analysed to check the quality and quantity of DNA. DNA was also quantified by agarose gel electrophoresis. 10 μl of diluted DNA (10-50 ng) was resolved on 0.8% TAE agarose gels (containing a 10^-5 ^dilution of Gel Red; Biotium) by electrophoresis at 100 v for 60 min, and visualized by UV fluorescence using an Ingenius Gel Documentation System (Syngene). Light densities from the resolved DNA samples were quantified by comparing amplified product UV intensity against standard amounts of titrated λ-DNA resolved in the same way.

#### Gel analysis of amplified products

After amplification, samples were routinely resolved on 2% TAE agarose gels (containing 10^-5 ^volumes of gel red; Biotium) at 100 volts for 60 min. The resolved amplicon was visualized and photographed over UV light, using an Ingenious Gel Documentation System (Syngene).

#### Copy Number Estimation

Target gene/transgenic element copy number was estimated by calculation assuming: the length of the maize genome [38]; 2 (diploid) copies of target polynucleotide/extracted genome; the average weight of a base pair (bp) is 650 Daltons; each bp has the same mass; the inverse of the calculated molecular weight is equivalent to the number of moles per gram and that using Avogadro's constant (6.022 × 10^23^) gives the copies of template/gram sample http://www.uri.edu/research/gsc/resources/cndna.html

$$Copies\;of\;target\;per\;genome=\left(\text{ng double stranded DNA}\right)\times \left(6.022\times 10^{23}\right)/\left(\text{length in bp }\times 10^{9}\times 650\right)\times 2$$

#### LAMP-BART reaction mixture

LAMP-BART reactions were performed in a total volume of 20 μl. A LAMP-BART master mix that contained 1.6 μM of each LAMP primer, 0.8 μM each loop primer and 0.4 μM of each displacement primer, 300 μM each dNTP (Invitrogen), 87 mM trehalose (Sigma), 10 mM DTT (Sigma), 3.5 mM luciferin (Europa Bioproducts Ltd), 250 μM APS (Biolog Institute), Ultraglow Luciferase (Promega; 5.6 μg/ml), ATP sulphurylase (NEB; 375 milliunits/ml), 6.4 U Bst polymerase (NEB), PVP (Sigma; 0.4 mg/ml), 60 mM KCl (Sigma), 2 mM MgSO_4 _(NEB), diluted in the required amount of Thermopol buffer (NEB). Each reaction was made up to volume by adding the specified amounts of the target DNA or molecular grade water.

#### BART analysis

All LAMP-BART coupled amplifications were performed on dedicated instruments that simultaneously control temperature and quantify bioluminescence during a given assay. Two variations of the hardware were used; a static thermally controlled machine, equipped with a charged coupled device camera http://www.lumora.co.uk, that has no theoretical limit of sample numbers or configurations; and a portable device (19; photodiode quantification PDQ; http://www.lumora.co.uk), that quantifies light using photo-diodes, which is presently limited to the analysis of 16 samples. All LAMP-BART reactions were performed in suitable nuclease free plastic tubes under molecular grade mineral oil, at 60°C for 90 min.

#### RT-PCR analysis

Each 25 μl PCR reaction was performed using the JumpStart SYBR Green ready mix (Sigma) supplemented with 5 pmol of respective primers (a dedicated pair for each target; Table 1). Reaction mixtures were denatured for 2 min at 94°C (to disassociate the polymerase from its protective antibody). Each cycle was: 94°C for 30 s, 50°C for 30 s, 72°C for 30 s, for 40 cycles. Amplification and analysis was performed using an ABI Prism 7000 sequence detection system (Applied Biosystems). Results were processed using Applied Biosystems SDS 2.312 software.

**Table 1:** Details of the primers used in the LAMP-BART and RT-PCR amplifications

Primer Type	Orientation	Target (5' base)	Primer Sequence (5'-3')
Displacement	sense	ADH1 (7)	CTTTGGATCGATTGGTTTC

Displacement	antisense	ADH1 (287)	CCCAAAATTACTCAACG

LAMP	sense	ADH1 (116)	GGTGATCAAGTGCAAAGGTCTTTTCATAAACCAAGATTAGTCAGATCAAG

LAMP	antisense	ADH1 (94)	CCCCTCCGCAAATCTTCGAACAGTTTTGTAACTGGTGAAGGACTGAG

LOOP	sense	ADH1 (68)	CGCCTTGTTTCTCCTCTGTC

LOOP	antisense	ADH1 (136)	CCAAATCATCCACTCCGAGAC

Displacement	sense	CaMV-35 S-p (7214)	AGGAAGGGTCTTGCG

Displacement	antisense	CaMV-35 S-p (7404)	ATAAAGGAAAGGCCATCG

LAMP	sense	CaMV-35 S-p (7317)	GTCTTCAAAGCAAGTGGTTTTGGATAGTGGGATTGTGCG

LAMP	antisense	CaMV-35 S-p (7296)	TTCCACGATGCTCCTCGTTTTCCTCTGCCGACAGTGG

LOOP	sense	CaMV-35 S-p (7274)	TCCACTGACGTAAGGG

LOOP	antisense	CaMV-35 S-p (7350)	GGGGTCCATCTTTGGG

Displacement	sense	NOS-t (1850)	CGCGATAATTTATCCTAGTTTG

Displacement	antisense	NOS-t (2053)	CGTTCAAACATTTGGCAAT

LAMP	sense	NOS-t (1962)	GCATGACGTTATTTATGAGATGGGTTTTCGCTATATTTTGTTTTCTATCGCG

LAMP	antisense	NOS-t (1947)	CATGCTTAACGTAATTCAACAGTTTTTGAATCCTGTTGCCGGTC

LOOP	sense	NOS-t (2007)	GATTAGAGTCCCGCAATTATAC

LOOP	antisense	NOS-t (1925)	AAATTATATGATAATCATCGCAA

PCR	sense	ADH1 (1297)	AATTTTGGGGAAAGCTTCGT

PCR	antisense	ADH1 (1369)	TTCACCACGATTGCAGGATA

PCR	sense	CaMV-35 S-p (7133)	GATTCCATTGCCCAGCTATC

PCR	antisense	CaMV-35 S-p (7215)	CAACGATGGCCTTTCCTTTA

PCR	sense	NOS-t (1854)	TCGTTCAAACATTTGGCAAT

PCR	antisense	NOS-t (1885)	AAGACCGGCAACAGGATTC

Underscored bases of the LAMP primers are additional foreign nucleotides, introduced to link different homology segments (CAMV-35 S-p LAMP primers contain four linker bases, while the other LAMP primers only contain 3 linker bases)

#### Primer design and synthesis

Previously published LAMP primers [39], (Table 1) were used to target the cauliflower mosaic virus 35 S promoter (CaMV 35 S-p; GenBank: X79465), and the *Agrobacterium tumefaciens *nopaline synthetase gene terminator (NOS-t; GenBank: V00087; 41), while the LAMP primers used to target *Zea mays *alcohol dehydrogenase reference gene (ADH1; GenBank: NM_001111939) were designed according to http://loopamp.eiken.co.jp/e/lamp/primer.html. The same three genes were also amplified by PCR (see Table 1); these primers were designed using Primer 3 http://frodo.wi.mit.edu/primer3/. All the primers were synthesisized by Eurofins MWG Operon as desalted, unmodified deoxribonucleotide oligonucleotides.

#### Regression analysis

Regression analysis was performed on experimental data sets where amplification procedures were assessed against various titrations of maize genomic DNA extracted using the 3 commercial extraction techniques and Lumora's simplified technique described above. The variation, linearity and efficiencies of the amplifications were calculated according to the mathematical algorithms stipulated by Pfaffl [40].

#### Comparative inhibition

Bt11 maize genomic DNA samples (5% GM; comprising 10^5 ^and 5 × 10^3 ^copies of wt and GM genomic copies respectively) prepared using Promega's Genome Wizard Kit were subjected to RT-PCR and LAMP amplifications as described earlier in the presence or absence of the following concentration of inhibitors (introduced into each assay at the following concentrations): SDS (sodium dodecyl sulphate; (0.005%; 0.01%; w/v); CTAB (cetyl trimethyl amonium bromide; 0.005%; 0.01%; w/v); NaCl (sodium chloride; 25 mM; 50 mM); Xylan (0.1%; 0.25%; w/v); Starch (0.1%; 0.25%; w/v); Humic Acid (0.01 ng; 0.1 ng); CaCl_2 _(1 μM; 100 μM).

#### Effect of carrier DNA on low copy amplification

The maize reference materials (0.1%, 0.5%, 1%, 2% and 5% GM), were extracted using Promega's Genome Wizard kit according to the manufacturer's instructions. The final air-dried pellets were hydrated in TE buffer and stored at 4°C. Extracted maize genomic DNA was quantified using the Nano Drop and electrophoresis methods described above. DNA from each extracted reference sample equivalent to 300, 200, 100 or 50 copies of 35 S promoter were assayed using the respective LAMP-BART assay to determine whether the genomic load has an impact upon the kinetics, reproducibility or sensitivity of this amplification method.

## Results & Discussion

### DNA extraction procedure affects quality estimations

**Figure 2:**
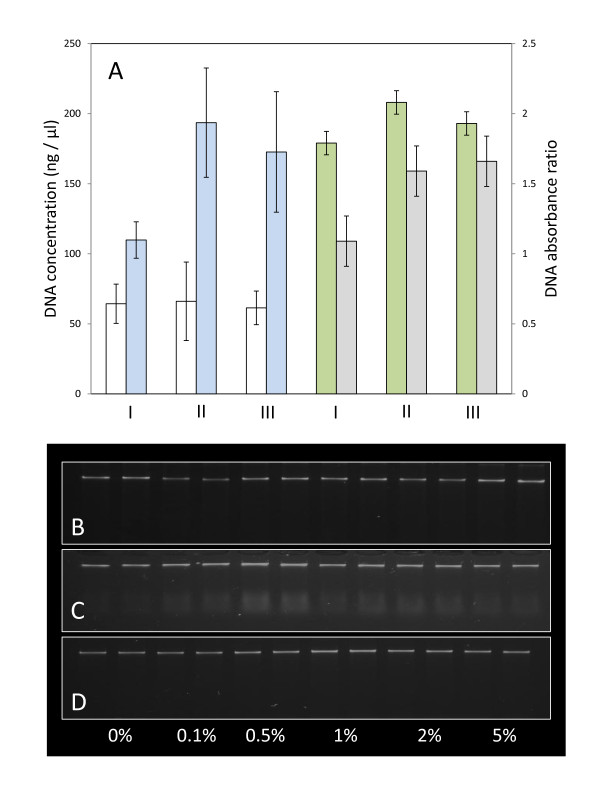
**Estimation of genomic DNA quantity and quality**. Bar graph represents average estimated DNA concentrations from at least 50 extracted samples (**A**; ng/μl), as defined by gel density analysis (white bars), or spectrophotometric determinations using a NanoDrop spectrophotometer (blue bars). Results were obtained for DNA samples extracted using Promega's Genome Wizard kit (**I**), the Nucleon Phytopue Kit (**II**) or the CTAB-homebrew method (**III**). Collated absorbance ratios obtained from the same samples at 260: 280 nm (green bars), or 260: 230 nm (grey bars) are shown. DNA quantity and integrity was assessed by resolving DNA samples (extracted from Bt11 maize reference material; 0%, - 5%) on 0.8% TAE agarose gels by electrophoresis. Representative samples extracted using Promega's Genome Wizard kit (**B**), the Nucleon Phytopue Kit (**C**) or the CTAB-homebrew method (**D**).

The quality of maize genomic DNA samples, extracted from Bt11 seed reference material were compared after extraction, using three different and commonly used plant DNA extraction methods (CTAB; Nucleon Phytopure™; Promega Genome Wizard™). When DNA was extracted using the Nucleon Phytopure kit, low molecular weight contamination was observed on gels after resolution of extracted samples by electrophoresis. Gel analysis of comparative DNA samples, extracted using the other methods, revealed little or no contamination (Figure 2B, C &2D). All three extraction methods yielded DNA apparently suitable for both PCR and LAMP-BART analysis, as defined by absorbance ratio (Figure 2A). We noted that DNA quantification was dramatically influenced by the choice of extraction and quantification technique. Gel images of the Nucleon Phytopure kit show fluorescence below the high molecular weight band, and may be consistent with RNA contamination (Figure 2C). RNA contamination may affect quantification, which is markedly higher (2 fold) when extracts were assessed using the spectrophotometric method (NanoDrop), compared to those made by gel density estimations. However, this discrepancy in calculated DNA yield was less obvious in samples of DNA extracted using the Promega Genome Wizard kit. Given potential differences in sample purity and composition, we suggest that the gel density method is likely to be a more reliable indication of comparative genomic DNA yield than spectrometry.

### Detection of maize GM Bt11 event using LAMP-BART and effect of DNA extraction procedures

**Figure 3:**
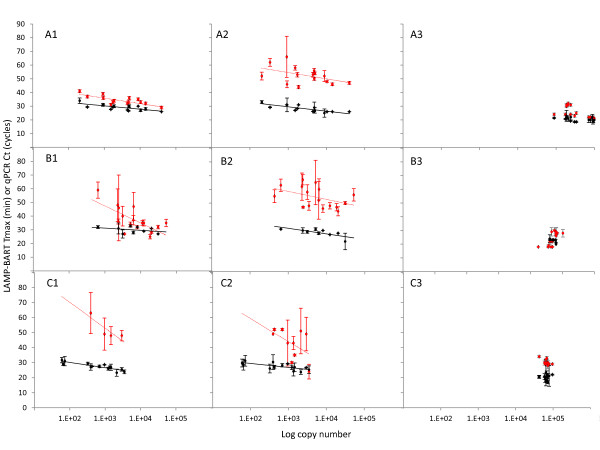
**Influence of the DNA extraction method on RT-PCR and LAMP-BART efficiency**. LAMP-BART (red points) and RT-PCR (black points) standard curves made by serial dilutions of DNA isolated using Promega's Genome Wizard kit (**A**), the Nucleon Phytopure Kit (**B**) or the CTAB-homebrew method (**C; **diluted 10 fold to reduce inhibitory nature of this preparation). DNA isolated using each extraction technique was amplified with the CaMV 35 S-p (1), NOS-t (2) or ADH1 (3) primer sets. Template concentration in LAMP-BART comparisons are plotted as a function of time to light peak (T_max_), while RT-PCR comparisons are based on cycle number (Ct). Each RT-PCR cycle is approximately equivalent to 2 min.

A comparison of LAMP-BART and PCR techniques was made using a titration series of maize Bt11 reference genome samples containing certified proportions of the Bt11 genome in a background of non-transgenic maize (0, 0.1, 0.5, 1.0, 2.0 & 5.0%), extracted from maize meal powder using three commercial genomic DNA extraction procedures. In each case, the total DNA load per assay remained constant, while GM copy number varied (50 to 10^3 ^copies/reaction) due to the different GM proportions in the original samples, from which the DNA was extracted. Both the GM LAMP-BART and RT-PCR techniques developed for this investigation were sufficiently sensitive to amplify reproducibly the cauliflower mosaic virus (CaMV) 35 S gene promoter (35 S-p) and nopaline synthase terminator (NOS-t) sequences, present in the transgene of the GM component of the reference samples, containing only 0.1% Bt11 maize powder. Both techniques amplified the endogenous ADH1 reference gene reproducibly for a given genome copy number (Figure 3).

Each set of GM-LAMP-BART and RT-PCR assays was shown to be linear with respect to GM copy number, with comparable velocities (each Ct value presented represents 2 minutes). The linear regression analysis, demonstrated major differences in observed velocity, sensitivity and reproducibility for each LAMP-BART assay, which was largely extraction dependent (Figure 3). Assays exhibited greater variability and a reduced sensitivity when DNA was extracted using either the CTAB or Phytopure chemistries (Figure 3B and 3C). DNA extracted with the Promega Genome Wizard procedure had little impact upon either amplification technology (Figure 3, Table 2), and greater sensitivity and more reproducible results were achieved.

**Table 2:** Regression analysis was performed on PCR and LAMP-BART data sets obtained using various DNA extractions performed on a titration series of Bt11 maize reference tissue (0.1 - 5%; nd - not determined)

Target	Amplification	Extraction	Slope	Lowest detectable	Amplification
**Sequence**	**Technique**	**Technique**	**(R^2^)**	**copy N^o ^(0.1% GM)**	**Efficiency *E *(+/- SD)**

CaMV-35 S-p	PCR	Promega Wizard	0.522	205	2.64 (0.46)

CaMV-35 S-p	PCR	Nucleon Phytopure	0.131	438	2.32 (0.08)

CaMV-35 S-p	PCR	CTAB (10^-1^)	0.495	70	2.47 (0.82)

CaMV-35 S-p	PCR	Lumora's Simplified	*nd*	*nd*	*nd*

NOS-t	PCR	Promega Wizard	0.657	205	2.16 (0.41)

NOS-t	PCR	Nucleon Phytopure	0.471	438	2.13 (0.18)

NOS-t	PCR	CTAB (10^-1^)	0.775	70	1.91 (0.19)

NOS-t	PCR	Lumora's Simplified	*nd*	*nd*	*nd*

CaMV-35 S-p	LAMP-BART	Promega Wizard	0.565	205	1.55 (0.22)

CaMV-35 S-p	LAMP-BART	Nucleon Phytopure	0.471	641	1.24 (0.13)

CaMV-35 S-p	LAMP-BART	CTAB (10^-1^)	0.247	350	1.1 (0.03)

CaMV-35 S-p	LAMP-BART	Lumora's Simplified	0.403	40	1.21 (0.07)

NOS-t	LAMP-BART	Promega Wizard	0.215	205	1.70 (0.73)

NOS-t	LAMP-BART	Nucleon Phytopure	0.196	438	1.31 (0.33)

NOS-t	LAMP-BART	CTAB (10^-1^)	0.740	350	1.15 (nd)

NOS-t	LAMP-BART	Lumora's Simplified	0.119	40	1.25 (0.07)

In all sets of analysis undertaken by RT-PCR, CaMV 35 S-p was amplified more efficiently than NOS-t, whereas efficiencies of amplification obtained using LAMP-BART were similar regardless of target sequence. However RT-PCR had a lower threshold of detection than LAMP-BART more reproducible data was achieved using the RT-PCR technique regardless of the extraction adopted in this experiment (Figure 3).

These results indicate that the DNA extracted using the CTAB and Phytopure methods are higher in contaminants or sample impurities incompatible with the LAMP-BART chemistry, perhaps reflecting their original development to service PCR. LAMP-BART and RT-PCR amplifications may therefore be affected by different inhibitors, either derived from the sample or the extraction procedure. The observed differences in the quantification data discussed previously (Figures 2 and 3) would lend weight this hypothesis. We therefore assessed the response of LAMP-BART to classical PCR inhibitors.

### Inhibitors of RT-PCR and LAMP-BART

The effect of a range of inhibitors known to affect Taq polymerase [25,27] was tested on LAMP-BART to assess whether they affect the Bst polymerase used in LAMP or the LAMP-BART reaction couple. Comparative assessment of RT-PCR and LAMP-BART kinetics was therefore carried out in the presence of known PCR inhibitors. Promega Wizard extracted Bt11 maize genome was used during this investigation to standardize the GM target in each assay (5% maize reference genome; 10^5 ^copies ADH or 10^3 ^copies of the GM targets per assay). Moreover, the previous experiments confirmed that the DNA extracted using this technique was likely to contain fewer impurities and consequently, the lowest innate influence over either amplification methods (Figure 3, Table 2).

**Figure 4:**
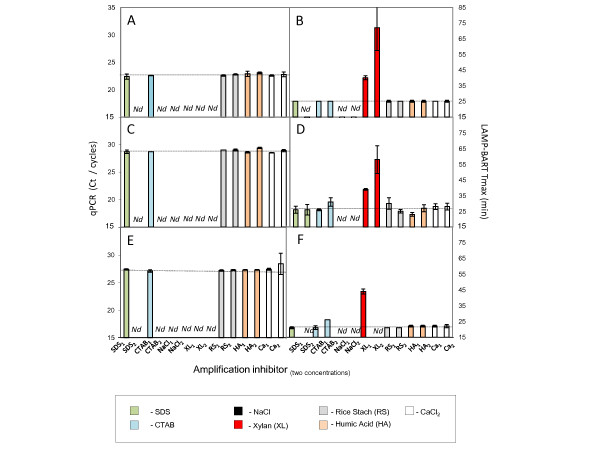
**Comparison of inhibitors of PCR and LAMP-BART**. Bt11 maize genomic DNA samples (5% GM) were subjected to RT-PCR amplification using ADH1 primers (A); CaMV 35 S-p primers (C) or NOS-t primers (E). A comparative set of analysis was performed using LAMP-BART, conducted in the presence of ADH1 primers (B); CaMV 35 S-p primers (D) or NOS-t primers (F). The extracted sample contained either 10^5 ^copies of wild type genome or 5 × 10^3 ^copies of transgenic genome. The analysis was performed in the presence and absence of final inhibitor concentration defined: sodium dodecyl sulphate; (SDS; 0.005% [1]; 0.01% [2] – GREEN BARS); cetyl trimethylamonium bromide (CTAB; 0.005% [1]; 0.01% [2] – BLUE BARS); sodium chloride (NaCl; 25mM [1]; 50mM [2]- BLACK BARS); xylan (XL; 0.1% [1]; 0.25% [2] – RED BARS); rice starch (RS; 0.1% [1]; 0.25% [2] – GREY BARS); humic acid (HA; 0.01ng [1]; 0.1ng [2] – BROWN BARS); calcium chloride (CaCl_2_; (1µM [1]; 100µM [2] – WHITE BARS). The broken line (----------), represents the average Ct or Tmax value for each set of amplifications performed in the absence of inhibitor. In some instances amplification or reporting of this activity was completely inhibited (not determined; *Nd*).

The monovalent salt (NaCl) abolished both types of amplification at 25 mM, regardless of the target and template concentration (Figure 4). This salt is likely to be perturbing these amplifications *via *Cl^-^, as this anion is known to compete for the active site of polymerase enzymes more effectively than phosphate, glutamate and acetate [41]. Clearly both polymerase enzymes are susceptible to this type of inhibition. The RT-PCR reaction was also strongly inhibited by the higher concentration of SDS and CTAB used (0.01%), and 0.1% of the acidic polysaccharide xylan. These inhibitors had little or no effect upon the kinetics of the equivalent LAMP-BART reactions (Figure 4B, D, F), although these detergents did reduce the output light intensity of the BART reporter, and hence were probably affecting one or more of the enzymes used to generate the light signal rather than LAMP amplification (19; data not shown). Neither starch, humic acid nor CaCl_2 _affected the kinetics of either amplification technique (Figure 4), at the concentrations used. It is also notable that RT-PCR reactions were either apparently unaffected or completely inhibited by specific inhibitors, and these effects are common to the different primer pairs tested. In contrast, LAMP-BART showed differences between the responses of different primer sets and also displayed increased reaction times in the presence of certain inhibitors particularly xylan. This points to the need for appropriate controls for sample inhibition in quantitative assays.

These results indicate that *Taq *polymerase is more prone to inhibition by plant acidic polysacharrides than *Bst *polymerase. It is therefore likely that more rapid solutions for DNA extraction may be appropriate for isothermal amplifications from matrixes that utilize these displacement polymerases as will be demonstrated later in this manuscript.

### The effect of total DNA concentration on LAMP-BART

**Figure 5:**
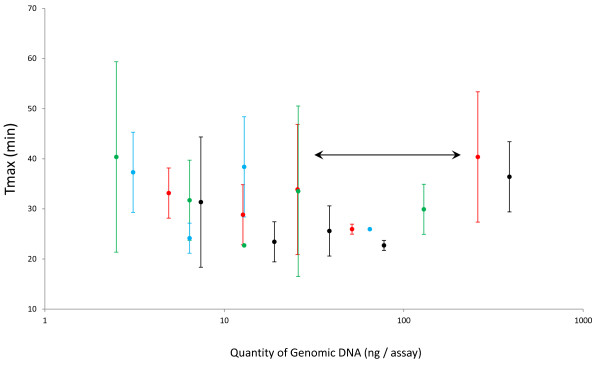
**Effect of varying total DNA concentration on CaMV 35 S-p LAMP-BART assay kinetics and reproducibility**. Graph of average Tmax values obtained for a constant target copy number present in a range of total maize genomic DNA concentrations, for each of 4 target copy numbers: 300 copies/assay (black circle symbol); 200 copies/assay (red circle symbol); 100 copies/assay (green circle symbol) and 50 copies/assay (blue circle symbol). Errors represent standard deviation of the mean (three replicates). The least variability in Tmax data was observed between the concentrations of genome marked with an arrow (50-500 ng).

A further contributor to the sensitivity of molecular amplifications is the total quantity of DNA in the reaction, as this is thought to affect the retention of polynucleotides to plastic ware, and sequester primers and/or polymerase, and can thereby reduce mis-amplification events [36,42]. We therefore investigated the reproducibility of LAMP-BART assays across a wide range of total DNA concentration from 5-750 ng per assay containing a constant amount of the target sequence. Results were determined for 50, 100, 200 and 300 copies of target. The data (Figure 5) show that reproducibility of the CaMV 35 S-p LAMP-BART significantly deteriorates when the total amount of genome is below 50 ng or above 500 ng total assay DNA, regardless of the target copy number; the converse is apparent when the target polynucleotides are assayed between these copy numbers (Figure 5), where a much higher degree of reproducibility is achieved.

LAMP-BART quantification relies on dependable assay kinetics and accurate estimations of the ensuing time-to-maximum light output (Tmax; 19). These data show that the total DNA load within a given reaction must therefore be taken into consideration. Similar considerations apply when preparing standards for reference curves [43]. In the case of PCR, reports in the literature are sparse, but a few demonstrate the potential for carrier DNA to positively impact upon the sensitivity and specificity of low copy PCR [36,43]. However, this is not observed when a hot start method is adopted [42]. It is thought that the carrier DNA affects the dominance of side reactions that occur before thermal cycling commences, while reactions are being formulated on the bench. If the polymerase is active during this phase in the procedure, then it can potentially propagate primer oligomerization and mis-priming events. Carrier DNA is thought to quench these side reactions at limited copy numbers, by sequestering the DNA polymerase and primers [36,44]. It is clear that the effect of carrier DNA is not limited to PCR, and is likely to be more significant in isothermal reactions, such as LAMP, where multiple primers are used to drive the amplification process.

Other reports describe how large amounts of non-target DNA can become limiting at the threshold of PCR detection, affecting both sensitivity and analytical kinetics [44], as we observed here (Figure 5). It is likely that higher genome loads and DNA concentrations compete for the DNA polymerase and primers intended for the target nucleotide. Together, this data defines a requirement to keep the carrier DNA within a given window for quantitative analysis. Furthermore, both biological samples and standards should be compared at equivalent concentrations of DNA.

### Rapid fieldable DNA extraction procedure

**Figure 6:**
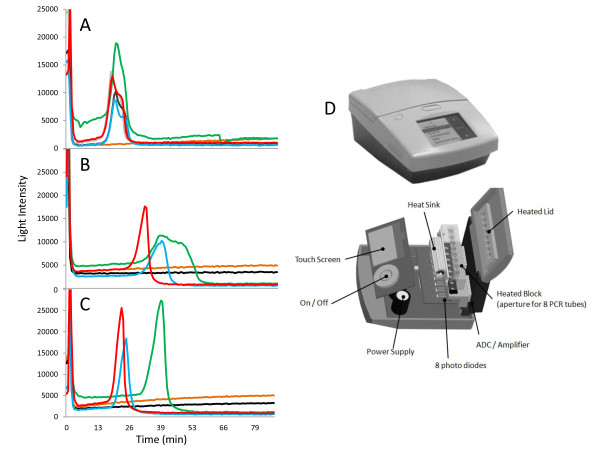
**Qualitative determinations of GM maize presence using fieldable extraction and amplification**. LAMP-BART light intensities recorded over time in amplifications of ADH1 (**A**), CaMV 35 S-p (**B**) and NOS-t (**C**), performed on DNA extracted from maize reference samples, containing either containing either 0% (brown line), 0.1% (green line), 1.0% (blue line) or 5.0% (red line) genetically modified maize or a MGW control (black line). The analysis was performed in a field environment using a portable instrument (PDQ; Lumora Ltd, Ely, UK); the exploded view illustrates the simplicity of this device (**D**).

A rapid and simple extraction technique was devised that capitalized on the increased tolerance of LAMP to acidic polysaccharide inhibition. Maize leaf discs (wt or Mon810) were extracted in sodium chloride (cell lysate) and CHELEX, a resin with a high affinity for divalent cations, thereby reducing problematic tertiary DNA structures that are known to become exacerbated in the presence of both Mg^2+ ^and Ca^2+^, and limiting DNA degradation after cell lysis by inhibiting DNase activity, as this enzyme requires Mg^2+ ^[45-49]. The resulting extracts were used successfully in LAMP-BART assays (Figure 6). Interestingly, the same samples were completely inhibitory to RT-PCR, known to be more prone to inhibition by acidic polysaccharides, which is a contaminant likely to be present in these plant extracts.

This simple genome extraction method allowed LAMP-BART amplification to be performed on 0.1% Bt11 maize reference sample (Figure 6A, B &6C). The amplification of both CaMV 35 S-p and NOS-t were shown to be linear with respect to target copy (data not shown) and achieved similar efficiencies and thresholds of detection compared to the same assays performed on DNA extracts obtained using commercial DNA extraction kits. Moreover, this extraction technique is suited to qualitative field testing, as it is rapid and only requires simple hardware (Figure 6D).

## Conclusions

Here we show the use of the recently described bioluminescent coupling of loop mediated amplification (LAMP) to the real-time bioluminescent reporting of amplification (BART) for the detection of low levels of genomic GM maize DNA, equivalent to contamination of 0.1% or 50 copies of GM target per 20 μl assay. The optimum level of total DNA in such LAMP-BART assays was determined to be in the range 75 ng per reaction (4 ng/μl). The sensitivity and reproducibility of reactions where GM target is limiting can be improved if the carrier DNA is supplemented to 80 ng/assay.

This investigation also highlights the impact that the choice of plant DNA extraction and quantification technique has on RT-PCR and LAMP-BART. The latter is less well suited to some conventional plant DNA extraction procedures, but is less affected by classical PCR amplification inhibitors, particularly acidic polysaccharides. A consequence of this robust nature was LAMP-BART's ability to amplify target DNA from rapidly extracted crude genome samples which were refractory to RT-PCR, and we show the application to a field-based qualitative test for GM maize. Together these data illustrate the potential for rapid testing of GM samples using LAMP-BART and highlights the importance of extraction technique, DNA quality and yield, genome and inhibitor loading on the quantitative nature of molecular tests.

## Competing interests

LT and JM are the inventors of Bioluminescent Assay in Real-Time (BART). They filed a patent and co-founded Lumora Ltd, a company that has full rights for the use of the BART patent. JM LT and OG declare financial interests in Lumora Ltd.

## Authors' contributions

The following authors were all employees at Lumora Ltd: GK OG CP CJM RJ NA CM and LT. GK PH NB OG CP CJM MR RJ NA CM conceived and designed the experiments detailed in this manuscript, while the experimental work was performed by GK PH NB MR OG, and the data analyzed by GK PH NB OG LT JAHM. The reagents, materials, and analytical tools were provided by GK PH NB OG NA LT JAHM and the manuscript was written by GK OG NA LT JAHM; all other authors have read and approved the final version of this manuscript.
